# Bioreactance reliably detects preload responsiveness by the end-expiratory occlusion test when averaging and refresh times are shortened

**DOI:** 10.1186/s13613-021-00920-7

**Published:** 2021-08-28

**Authors:** Francesco Gavelli, Alexandra Beurton, Jean-Louis Teboul, Nello De Vita, Danila Azzolina, Rui Shi, Arthur Pavot, Xavier Monnet

**Affiliations:** 1 Service de Médecine Intensive-Réanimation, Université Paris-Saclay, AP-HP, Hôpital de Bicêtre, DMU CORREVE, Inserm UMR S_999, FHU SEPSIS, CARMAS, 78, Rue du Général Leclerc, 94 270 Le Kremlin-Bicêtre, France; 2 Emergency Medicine Unit, Department of Translational Medicine, Università degli Studi del Piemonte Orientale, 28100 Novara, Italy; 3 Research Support Unit, Department of Translational Medicine, Università degli Studi del Piemonte Orientale, 28100 Novara, Italy

**Keywords:** Fluid, Cardiac index, Monitoring, Passive leg raising, Fluid challenge, Heart lung interactions

## Abstract

**Background:**

The end-expiratory occlusion (EEXPO) test detects preload responsiveness, but it is 15 s long and induces small changes in cardiac index (CI). It is doubtful whether the Starling bioreactance device, which averages CI over 24 s and refreshes the displayed value every 4 s (Starling-24.4), can detect the EEXPO-induced changes in CI (ΔCI). Our primary goal was to test whether this Starling device version detects preload responsiveness through EEXPO. We also tested whether shortening the averaging and refresh times to 8 s and one second, respectively, (Starling-8.1) improves the accuracy of the device in detecting preload responsiveness using EEXPO.

**Methods:**

In 42 mechanically ventilated patients, during a 15-s EEXPO, we measured ∆CI through calibrated pulse contour analysis (CI_pulse_, PiCCO2 device) and using the Starling device. For the latter, we considered both CI_Starling-24.4_ from the commercial version and CI_Starling-8.1_ derived from the raw data. For relative ∆CI_Starling-24.4_ and ∆CI_Starling-8.1_ during EEXPO, we calculated the area under the receiver operating characteristic curve (AUROC) to detect preload responsiveness, defined as an increase in CI_pulse_ ≥ 10% during passive leg raising (PLR). For both methods, the correlation coefficient *vs.* ∆CI_pulse_ was calculated.

**Results:**

Twenty-six patients were preload responders and sixteen non preload-responders. The AUROC for ∆CI_Starling-24.4_ was significantly lower compared to ∆CI_Starling-8.1_ (0.680 ± 0.086 *vs.* 0.899 ± 0.049, respectively; *p* = 0.027). A significant correlation was observed between ∆CI_Starling-8.1_ and ∆CI_pulse_ (*r* = 0.42; *p* = 0.009), but not between ∆CI_Starling-24.4_ and ∆CI_pulse_. During PLR, both ∆CI_Starling-24.4_ and ∆CI_Starling-8.1_ reliably detected preload responsiveness.

**Conclusions:**

Shortening the averaging and refresh times of the bioreactance signal to 8 s and one second, respectively, increases the reliability of the Starling device in detection of EEXPO-induced ∆CI.

*Trial registration:* No. IDRCB:2018-A02825-50. Registered 13 December 2018.

**Supplementary Information:**

The online version contains supplementary material available at 10.1186/s13613-021-00920-7.

## Background

Over the last decade, much effort has been put into the development of methods monitoring cardiac index (CI) non-invasively [1–6]. Among them, bioreactance estimates cardiac output by analyzing the phase shift between an inward current that is sent through the thorax and the resulting outward current [1]. The principle of the technique is that this phase shift is determined by the variation of the volume of the thorax. From beat to beat, this variation is related to the variation of the volume of blood in the descending aorta and, thus, to stroke volume [7]. Bioreactance is considered as an improvement of bioimpedance which might be less sensitive to artifacts and the patient’s movements. The technique is totally non-invasive, as it only requires electrodes pasted on the thorax.

It has been shown to detect real-time changes of CI (ΔCI) induced by a passive leg raising (PLR) test and volume expansion [7]. Besides the PLR test, the end-expiratory occlusion (EEXPO) test is another test assessing preload responsiveness which can be used in mechanically ventilated patients. It consists in interrupting mechanical ventilation at end-expiration for a few seconds, which increases cardiac preload, and in observing the ΔCI which occurs in cases of preload responsiveness. Its accuracy has been established [12–14] and it is easy to perform.

**Fig. 1:**
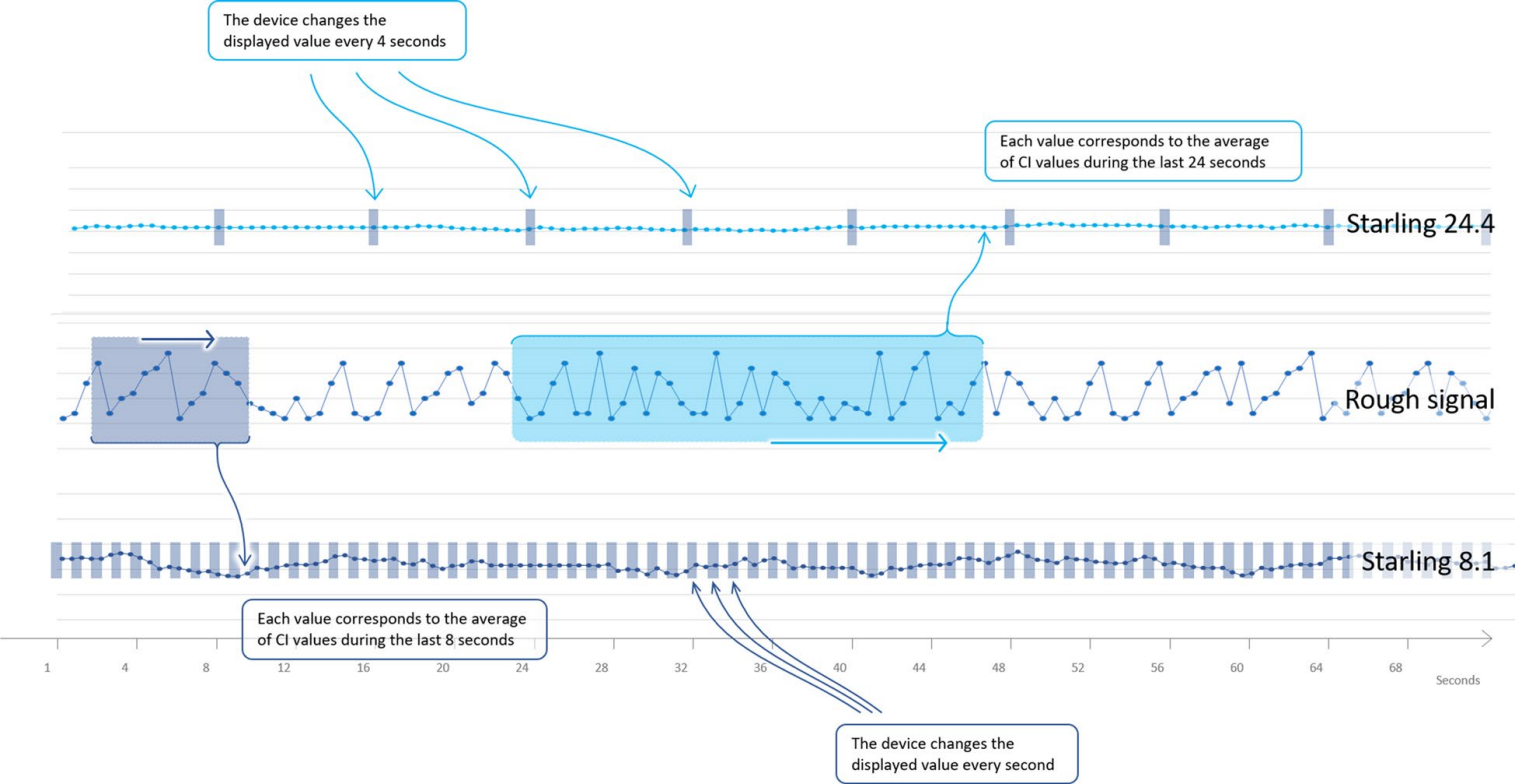
Averaging and refresh times of both the commercial (Starling-24.4—upper panel) and research (Starling-8.1—lower panel) bioreactance devices. CI: cardiac index; Starling-24.4: commercial version of the Starling device (averaging time 24 s, refresh time 4 s); Starling-8.1: research Starling device (averaging time 8 s, refresh time one second)

Nevertheless, the duration of EEXPO is only 15 s, and the induced ΔCI are relatively small [12]. It is then uncertain whether the available commercial version of the bioreactance device, which averages the CI signal over 24 s and refreshes the displayed value every 4 s, is adequate for monitoring the effects of EEXPO (Fig. 1). Thus, the primary goal of this study was to test whether the commercial version of the bioreactance device accurately detects preload responsiveness through the EEXPO-induced ΔCI. The secondary goal was to assess whether shortening the averaging and refresh times of the device improves this detection. We hypothesized that bioreactance can monitor the EEXPO effects on CI, provided that the time over which it averages CI and after which it refreshes its displayed value is short.

## Patients and methods

### Patients

This prospective study was conducted in a 25-bed intensive care unit (ICU) and approved by an Institutional Review Board (No. IDRCB: 2018-A02825-50). At the time of inclusion, patients’ next of kin were informed of the study protocol and of the option to refuse participation. As soon as clinical conditions improved and patients were able to give consent, the same opportunity was given to them. All patients and/or relatives agreed to participate.

Patients were included if they met the following inclusion criteria: age ≥ 18 years, admission to the ICU for less than 24 h, invasive mechanical ventilation, PiCCO2 device already in place (Pulsion Medical Systems, Feldkirchen, Germany) and decision by the attending clinicians to perform a PLR test. Exclusion criteria were intra-abdominal hypertension and venous compression stockings (which may decrease the PLR test reliability) [15], intracranial hypertension (which is a contraindication for PLR) and inability of the patients to sustain a 15-s EEXPO. Patients were included depending on the availability of the investigators. The study report complies with the Standards for Reporting Diagnostic Accuracy (STARD) statement [16].

### Bioreactance measurements

The Starling v5.5 device (Baxter, Deerfield, IL, USA) requires 4 double-electrode sensors pasted on the thorax skin, creating a “virtual box” around the heart. The upper sensors are placed on the mid-right and mid-left clavicles and the lower sensors on the mid-right and mid-left last ribs. In each electrode pair, the outer one delivers a current with known alternating high frequency, detected by the inner electrode pair. The phase modulation between currents recorded at the inner and outer electrodes is altered by the changes in thoracic pulsatile blood volume, which allows a proprietary algorithm to derive stroke volume and CI [1, 17, 18].

The Starling v5.5 device displays a CI value which corresponds to the moving average of the raw values that have been measured over the last 24 s (Fig. 1). The displayed average is refreshed on the screen every 4 s. The CI value measured in this way will hereafter be called “CI_Starling-24.4_”.

We also extracted raw data from our recordings by the Starling device. In a post-hoc analysis, we changed the averaging time to 8 s, instead of 24. This duration was the shortest possible time that could be achieved, according to the technological limitations of the currently available device. We judged this interval as appropriate for estimating the effects of the 15-s EEXPO.

The refreshing delay was reduced to one second, instead of 4. The CI value obtained in this way will be called “CI_Starling 8.1_” (Fig. 1).

### Transpulmonary thermodilution and pulse contour analysis measurements

The PiCCO2 device measures CI through transpulmonary thermodilution, which is performed by injecting three 15-mL boluses of cold saline in the superior vena cava [19, 20], and through pulse contour analysis (CI_pulse_), which is calibrated by transpulmonary thermodilution [21]. The value of CI_pulse_ provided by pulse contour analysis is averaged over 12 s, with values that are refreshed every second. CI_pulse_ was continuously recorded by the PiCCOWin software (Pulsion Medical Systems).

### Other measurements

In addition to arterial pressure, heart rate and CI, we measured central venous pressure at end-expiration. Respiratory variables such as positive end-expiratory pressure, plateau pressure, respiratory rate and tidal volume (Vt) were also collected. Intra-abdominal pressure was measured through the bladder pressure as previously described [22].

Arterial, central venous and airway pressures were continuously recorded by data acquisition software (HEM-3.5, Notocord, Croissy-sur-Seine, France).

### Study protocol

At baseline, a set of thermodilution measurements was performed and CI_pulse_ was calibrated. Once hemodynamic stability was observed (change in mean arterial pressure < 5% over 4 min) (EEXPO_start_), CI_pulse_, CI_Starling-24.4_, CI_Starling-8.1_ and other hemodynamic measurements were collected. A 15-s EEXPO was then initiated as previously described [12]. At the end of the EEXPO test (EEXPO_end_), the same variables were recorded. Subsequently, once the values of the hemodynamic variables had returned to baseline, another set of measurements were performed (PLR_start_). A PLR maneuver was performed as previously described [23], and, after 1 min of PLR, measurements were collected again (PLR_end_). If the ΔCI_pulse_ between PLR_start_ and PLR_end_ was ≥ 10%, the patient was defined as a “preload responder”. This threshold corresponds to the increase in CI that has been demonstrated to indicate preload responsiveness with the best combination of sensitivity and specificity [24]. Sedative drugs, catecholamines and ventilatory settings were kept unchanged during the study period.

### Statistical analysis

Based on a previous study by our group [11], to detect an increase in CI of at least 5% measured by the Starling device, expecting a baseline value of 3.1 L/min/m^2^, we estimated that 42 pairs of measurements were required. This assessment was performed taking into account an α risk of 5% and a *β* risk of 20%, estimating that half of the patients would be preload responders. The minimal change of 5% was chosen, because it corresponds to the best threshold of EEXPO-induced CI changes that detects preload responsiveness [14]. It is compatible with the least significant change of CI_pulse_ [25].

Data are summarized as mean ± SD or median [interquartile range, IQR] as appropriate. The normality of distribution was evaluated visually. Pairwise comparisons of data were done with the paired Student’s *t* test or Wilcoxon test. The two-tailed Student’s *t* test or Mann–Whitney *U* test compared preload responders and non-responders.

To assess the significance of changes of variables over time during different interventions, we used a linear mixed-effect model to evaluate the group (preload responders and non-responders) and time (EEXPO_start_, EEXPO_end_, PLR_start_, PLR_end_) effects on hemodynamic variables. Time and groups were assumed as fixed effects, also considering the interaction component. A random intercept term was considered in patients to account for correlation among repeated measurements. The post-hoc pairwise comparison was reported by adjusting *p* values for multiple testing, using the Holm method [26]. Regarding our primary goal, receiver operating characteristic curves for EEXPO-induced relative ∆CI_Starling-24.4_ to predict preload responsiveness were built, providing sensitivity, specificity and the best threshold, and their area under the receiver operating characteristic curve (AUROC) was measured. The same analysis was performed for ∆CI_Starling-8.1_ to assess our secondary goal, and the AUROC were compared with the Hanley–McNeil test [27]. The ability of both ∆CI_Starling-24.4_ and ∆CI_Starling-8.1_ to detect preload responsiveness was subsequently tested in the subgroup of patients with and without norepinephrine infusion and in patients with a high and a low body mass index (BMI). “High” and “low” BMI values were defined according to the median of the variable measured in the whole population. To evaluate the overall concordance between absolute values of CI_pulse_ and both CI_Starling 24.4_ and CI_Starling 8.1_ for EEXPO, we reported the intraclass correlation coefficient (ICC). Pearson’s correlation coefficient tested the correlations between the EEXPO-induced ∆CI_pulse_ and both ∆CI_Starling-24.4_ and ∆CI_Starling-8.1_, and these coefficients were compared for relative changes.

We compared the absolute values of CI_pulse_ and CI_Starling-24.4_ and the absolute values of CI_pulse_ and CI_Starling-8.1_ recorded during EEXPO_start_, EEXPO_end_, PLR_start_ and PLR_end_ using the Bland–Altman analysis. Limits of agreement plots were defined as accounting for repeated measurements with possibly heteroscedastic measurement errors [28]. A Critchley polar plot analysis was performed [29] for assessment of the trending ability of CI_Starling-24.4_ and CI_Starling-8.1_, to compare the concordance in terms of relative ∆CI_pulse_ *vs.* ∆CI_Starling-24.4_ and ∆CI_Starling-8.1_, both for EEXPO and PLR. Radial limits of agreement < 30° are considered to indicate good trending ability.

Statistical significance was set at a *p* value < 0.05 and statistical analysis was performed with MedCalc software 19.1 (Mariakerke, Belgium) and R 3.5.2 statistical software with lme4, MethodCompare and irr packages [30].

## Results

### Patients

Forty-two patients were included between April and September 2019. No patient was excluded due to inability to sustain a 15-s respiratory hold (Additional file 1: Figure S1). All patients were sedated with propofol and remifentanil (Table 1). Eight (19%) patients were paralyzed at the time of inclusion and no patient exhibited spontaneous breathing activity. No patient was in the prone position or had renal replacement therapy in place. Two patients had atrial fibrillation, whereas the others were in sinus rhythm (Table 1).

**Table 1:** Patient characteristics

Patient characteristics (*n* = 42)	
Age (years)	60 ± 9
Male gender (n, %)	21 (50%)
Body mass index (kg/m^2^)	24 [21–27]
Simplified Acute Physiologic Score II on inclusion	49 [31–55]
Richmond Agitation Sedation Scale score	− 5 [− 5 to − 4]
Left ventricular ejection fraction (%)	45 ± 6
Intra-abdominal pressure (mmHg)	13 ± 4
Type of shock (*n*, %)	
Septic Cardiogenic Hypovolemic Distributive non-septic	36 (85.7%) 4 (9.5%) 1 (2.4%) 1 (2.4%)
Atrial fibrillation (*n*, %)	2 (4.8%)
Cumulative fluid balance (mL)	1035 [734–1655]
ICU length of stay (days)	17 [7–44]
Mortality at day-28 (*n*, %)	13 (31%)
Norepinephrine	
Number of patients (%) Dose of norepinephrine (µg/kg/min)	27 (64%) 0.28 [0.13–0.43]
Ventilator settings	
Tidal volume (mL/kg of PBW) Respiratory rate (breaths/min) Fraction of inspired oxygen Positive end-expiratory pressure (cmH_2_O) Plateau pressure (cmH_2_O)	6.0 [5.1–6.0] 28 ± 5 0.51 ± 0.16 12 ± 3 25 ± 5

*ICU* intensive care unit, *PBW* predicted body weight

### Hemodynamic changes during interventions

Twenty-six (62%) patients were defined as preload responders, according to the results of the PLR test. The changes in hemodynamic variables in both groups are shown in Table 2.

**Table 2:** Hemodynamic measurements

Variables	EEXPO_start_	EEXPO_end_	PLR_start_	PLR_end_
Heart rate (min^−1^)				
Preload responders (*n* = 26)	95 ± 16	96 ± 17	96 ± 17	93 ± 18**
Preload non-responders (*n* = 16)	93 ± 23	93 ± 23	93 ± 22	93 ± 22
Systolic arterial pressure (mmHg)				
Preload responders (*n* = 26)	120 ± 17	121 ± 17	122 ± 20	136 ± 16**
Preload non-responders (*n* = 16)	134 ± 24^a^	134 ± 24^a^	132 ± 18	139 ± 18
Diastolic arterial pressure (mmHg)				
Preload responders (*n* = 26)	60 ± 11	60 ± 10	62 ± 11	67 ± 11**
Preload non-responders (*n* = 16)	68 ± 11^a^	68 ± 11^a^	67 ± 11	71 ± 9
Mean arterial pressure (mmHg)				
Preload responders (*n* = 26)	82 ± 12	82 ± 11	83 ± 13	93 ± 12**
Preload non-responders (*n* = 16)	92 ± 12^a^	92 ± 13^a^	91 ± 11^a^	96 ± 9
Central venous pressure (mmHg)				
Preload responders (*n* = 26)	11 ± 5	11 ± 4	12 ± 4	14 ± 5**
Preload non-responders (*n* = 16)	14 ± 4^a^	13 ± 4	14 ± 4	15 ± 3
PiCCO2 Cardiac Index (L/min/m^2^)				
Preload responders (*n* = 26)	2.95 ± 1.05	3.12 ± 1.06*	2.89 ± 0.94	3.40 ± 1.03**
Preload non-responders (*n* = 16)	3.03 ± 0.87	3.08 ± 0.89	2.97 ± 0.78	3.08 ± 0.89
Starling-24.4 Cardiac Index (L/min/m^2^)				
Preload responders (*n* = 26)	2.8 ± 0.5	3.0 ± 0.6*	2.8 ± 0.5	3.5 ± 0.7**
Preload non-responders (*n* = 16)	2.4 ± 0.4^a^	2.3 ± 0.4^a^	2.6 ± 0.5	2.6 ± 0.5^a^
Starling-8.1 Cardiac Index (L/min/m^2^)				
Preload responders (*n* = 26)	2.83 ± 0.58	3.25 ± 0.71*	2.69 ± 0.55	3.98 ± 0.86**
Preload non-responders (*n* = 16)	2.45 ± 0.41	2.48 ± 0.39^a^	2.63 ± 0.50	2.79 ± 0.51^a^
Pulse pressure variation (%)				
Preload responders (*n* = 26)	10 ± 6	–	11 ± 7	10 ± 6
Preload non-responders (*n* = 16)	10 ± 9	–	11 ± 8	10 ± 9
Stroke volume variation (%)				
Preload responders (*n* = 26)	12 ± 6	–	12 ± 6	11 ± 6
Preload non-responders (*n* = 16)	11 ± 8	–	11 ± 8	11 ± 8

^a^*p* < 0.05 *vs.* Preload responders

*p < 0.05* vs.* EEXPO_start_; **p < 0.05 *vs.* PLR_start_

PLR induced a ∆CI_pulse_ of 16.8 [12.0–24.43%] in responders and 2.2 [1.3–4.5%] in non-responders (*p* < 0.0001). It induced a ∆CI_Starling-24.4_ of 21.7 [14.3–43.8%] in responders and 0.0 [0.0–4.1%] in non-responders (*p* < 0.0001). PLR induced a ∆CI_Starling-8.1_ of 49.7 [29.3–74.4%] in responders and 5.1 [-0.4–11.1%] in non-responders (*p* < 0.0001) (Table 2).

The EEXPO test induced a ∆CI_pulse_ of 5.3 [4.1–7.5%] in responders and 1.2 [0.5–2.4%] in non-responders (*p* < 0.0001). It induced a ∆CI_Starling-24.4_ of 5.5 [− 0.2–7.1%] in responders and 0.1 [− 0.1–0.1%] in non-responders (*p* = 0.049). The EEXPO test induced a ∆CI_Starling-8.1_ of 12.8 [7.8–22.2%] in responders and 0.9 [− 1.1–4.8%] in non-responders (*p* = 0.0001) (Table 2).

### Ability of the EEXPO-induced ∆CI_Starling-24.4_ to detect preload responsiveness

The relative EEXPO-induced ∆CI_pulse_ detected preload responsiveness, as defined by a positive PLR test, with an AUROC of 0.983 ± 0.018. The cut-off corresponding to the best Youden index was 3.3% (Table 3).

**Table 3:** Ability of the end-expiratory occlusion test to detect preload responsiveness using three different methods for measuring cardiac index

Variable	AUROC ± SE	Sensitivity (95% CI)	Specificity (95% CI)	LR + (95% CI)	LR− (95% CI)	Cutoff	*p*
EEXPO—Relative ΔCI_pulse_	0.983 ± 0.018	1.00 (0.87–1.00)	0.94 (0.70–1.00)	16.0 (2.4–106.7)	–	3.3%	< 0.0001
EEXPO—Relative ΔCI_Starling-24.4_	0.680 ± 0.086	0.62 (0.41–0.80)	0.94 (0.70–1.00)	9.9 (1.4–67.3)	0.4 (0.2–0.7)	0.1%	0.036
EEXPO—Relative ΔCI_Starling-8.1_	0.899 ± 0.049	0.79 (0.59–0.93)	0.86 (0.57–0.98)	5.54 (1.5–20.3)	0.24 (0.1–0.5)	5.1%	< 0.0001

*AUROC* area under the receiver operating characteristic curve, *EEXPO* end-expiratory occlusion, *LR +*  positive likelihood ratio, *LR−* negative likelihood ratio, *PLR* passive leg raising; *SE* standard error, *95% CI* 95% confidence interval, *ΔCI*_*pulse*_ changes in cardiac index measured through the pulse contour analysis method, *ΔCI*_*Starling-24.4*_ changes in cardiac index detected by the commercial version of the Starling device (averaging time 24 s, refresh time 4 s), *ΔCI*_*Starling-8.1*_ changes in cardiac index derived through raw data analysis of the Starling device (averaging time 8 s, refresh time 1 s)

**Fig. 2:**
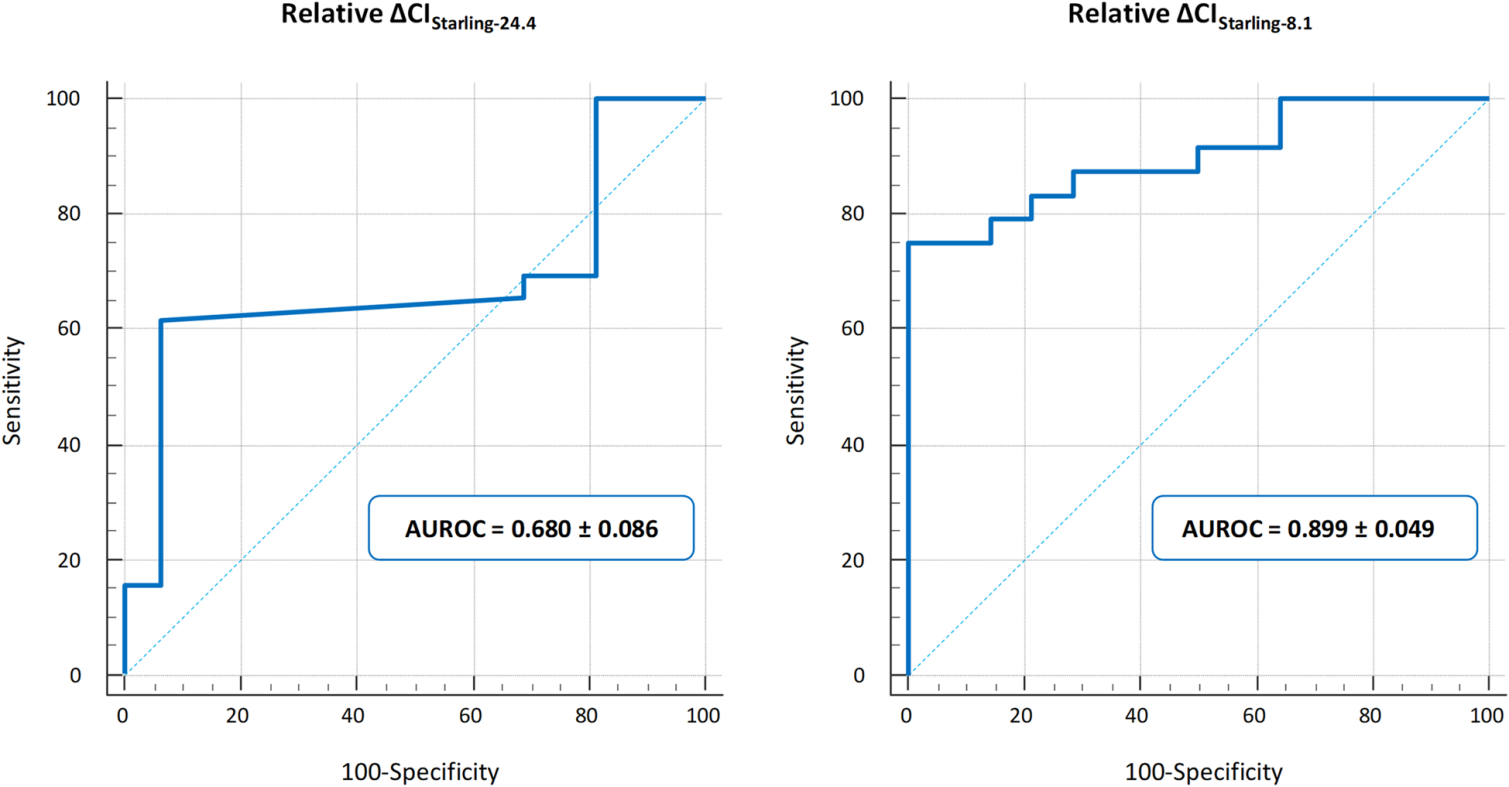
Ability of relative ΔCI_Starling-24.4_ (left) and ΔCI_Starling-8.1_ (right) to detect preload responsiveness at the EEXPO test. AUROC: area under the receiver operating characteristic curve; EEXPO: end-expiratory occlusion; ΔCI_Starling-24.4_: changes in cardiac index detected by the commercial version of the Starling device (averaging time 24 s, refresh time 4 s); ΔCI_Starling-8.1_: changes in cardiac index detected through raw data analysis of the Starling device (averaging time 8 s, refresh time one second)

The relative EEXPO-induced ∆CI_Starling-24.4_ detected preload responsiveness with an AUROC of 0.680 ± 0.086 and a best Youden index cut-off of 0.1% (Fig. 2, Table 3).

### Ability of the EEXPO-induced ∆CI_Starling-8.1_ to detect preload responsiveness

Relative EEXPO-induced ∆CI_Starling-8.1_ detected preload responsiveness with an AUROC of 0.899 ± 0.049 and a cut-off of 5.1% (Fig. 2, Table 3). The comparison with the AUROC of the EEXPO-induced ∆CI_Starling-24.4_ was significant (*p* = 0.027). At the EEXPO test, Starling-24.4 classified 10 patients as false negative and one as false positive, while Starling-8.1 classified 3 patients as false negative and 2 as false positive. When the same analysis was performed both in patients with and without norepinephrine infusion and in patients with high and low BMI, we observed similar results (Additional file 1: Tables S1 and S2).

### Ability of the PLR-induced ∆CI_Starling-24.4_ and ∆CI_Starling-8.1_ to detect preload responsiveness

Relative PLR-induced ΔCI_Starling-24.4_ detected preload responsiveness, as defined by the increase in ΔCI_pulse_ ≥ 10% during PLR, with an AUROC of 0.929 ± 0.039. The cut-off corresponding to the best Youden index was 10%. Similarly, PLR-induced relative ΔCI_Starling-8.1_ detected preload responsiveness with an AUROC of 0.970 ± 0.024 and a best Youden index cut-off of 15% (Additional file 1: Table S3).

### Concordance analysis

When considering all the changes observed during the study (n = 84) at Bland–Altman analysis, absolute values of both CI_Starling-24.4_ and CI_Starling-8.1_ showed a regressive pattern *vs. *CI_pulse_, with the bias line moving for higher values (Additional file 1: Figure S2). The percentage error was 67% for CI_Starling-24.4_ and 65% for CI_Starling-8.1_.

The ICC for absolute value comparison *vs.* CI_pulse_ at the EEXPO test (n = 42) was higher for CI_Starling-8.1_ than for CI_Starling-24.4_ (0.60 *vs.* 0.48, respectively; *p =* 0.04). Again, when considering only the changes observed during EEXPO, a significant correlation was observed between relative ∆CI_pulse_ and ∆CI_Starling-8.1_ (*r* = 0.42; *p = *0.009), but not between ∆CI_pulse_ and ∆CI_Starling-24.4_ (*p = *0.40). When considering only the changes observed during PLR, a significant correlation was observed both between ∆CI_pulse_ and ∆CI_Starling-8.1._ and between ∆CI_pulse_ and ∆CI_Starling-24.4_ (*r* = 0.70 and *r* = 0.60, respectively; *p *< 0.0001 for both).

**Fig. 3:**
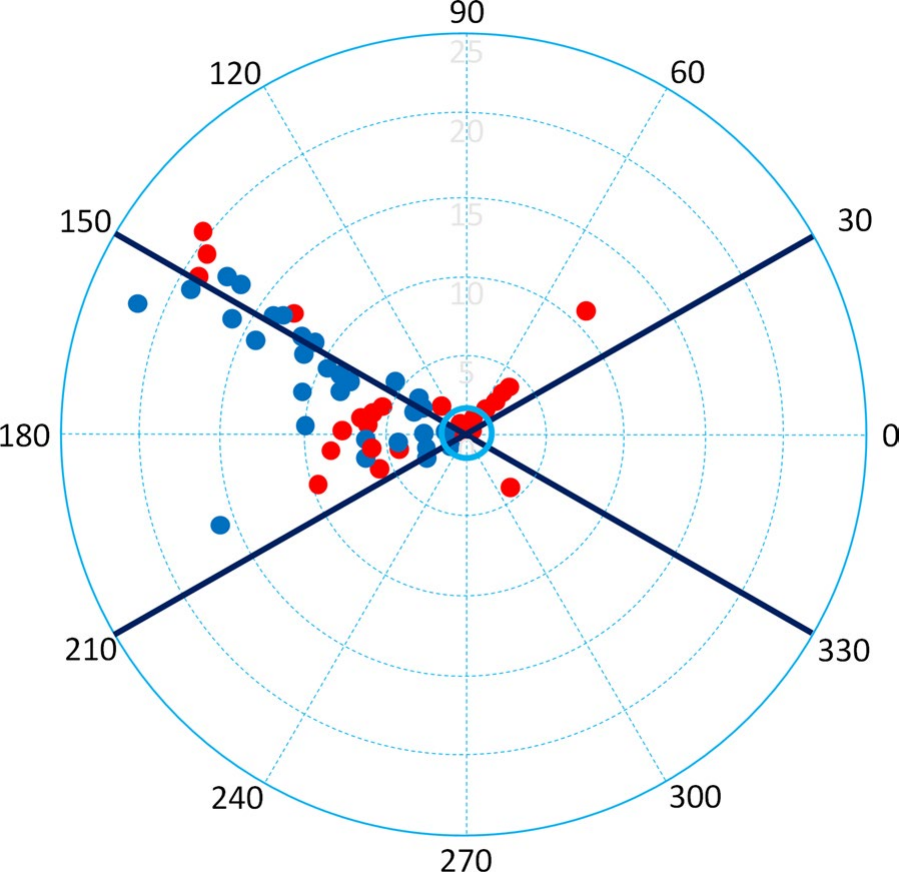
Polar plot analysis during the EEXPO test. Polar plot analysis for relative changes in CI_Starling-24.4_ (red dots) and CI_Starling-8.1_ (blue dots) compared to the changes in CI_pulse_ during the EEXPO test. The radial limits of the agreement have been reported. The central exclusion zone (continuous light blue circle) removes data points, where the changes in cardiac index are small (less than 1.5%). The polar concordance at 30 degrees is 83% for Starling-8.1.1 and 71% for Starling-24.4. CI_pulse_: cardiac index measured by the pulse contour analysis method; CI_Starling-24.4_: cardiac index measured by the commercial version of the Starling device (averaging time 24 s, refresh time 4 s); CI_Starling-8.1_: cardiac index derived through raw data analysis of the Starling device (averaging time 8 s, refresh time one second); EEXPO: end-expiratory occlusion

When considering only the changes observed during EEXPO (n = 42) at polar plot analysis, after removing from the central exclusion data points for which ΔCI were less than 1.5% [25], the ability to track ΔCI was higher for CI_Starling-8.1_ (polar concordance: 83%) than for CI_Starling-24.4_ (polar concordance: 71%) (Fig. 3). When considering only the changes observed during PLR (n = 42), the ability to track ΔCI was similar for CI_Starling-8.1_ (polar concordance: 81%) and for CI_Starling-24.4_ (polar concordance: 86%) (Additional file 1: Figure S3).

## Discussion

This study shows that the commercial version of the Starling device poorly detects preload responsiveness through the EEXPO test. However, when the hemodynamic effects of the EEXPO test are tracked with a modified version of the Starling device, where the averaging time is reduced to 8 s and the refresh time to one second, the ability to detect preload responsiveness is good. In addition, this study confirms that bioreactance reliably follows the PLR-induced ΔCI, whichever setting is used.

Over the years, different tests have been developed to detect preload responsiveness before deciding to infuse fluids or not [31]. However, these tests differ not only in the amplitude of ΔCI they induce, but also in the time over which these changes occur [13, 32]. In particular, the EEXPO test was performed over 12 to 30 s in the studies that tested its reliability [33, 34].

Regarding the different techniques estimating CI, the issue of averaging and refresh times is often neglected. Averaging the beat-to-beat values of CI allows the smoothing of CI changes, due either to its physiological instability or to the lack of precision of the technique that estimates it. Without any average, it would be difficult to distinguish small changes from the noise of the signal. Conversely, if the averaging period is very long, the signal could be so smoothed that small changes would be undetectable. Besides the averaging time, the frequency at which every new CI value is displayed is also crucial. If the value is refreshed at each cardiac beat, the displayed value may be very unstable, again impairing the assessment of significant changes. Conversely, in the event of infrequent refreshments, acute changes may be masked.

Our team has demonstrated that bioreactance did not reliably detect ΔCI induced by a 1-min PLR if the averaging time was 30 s [10]. A version of the NICOM device using a moving averaging period of 8 s was much better for this purpose [11]. In the present study, we investigated the ability of bioreactance to assess the EEXPO test, the duration of which is much shorter than that of the PLR test. For this purpose, we changed the averaging time and the refresh time from the raw values of CI estimated by bioreactance.

Regarding our primary goal, the EEXPO test was unable to detect preload responsiveness if assessed with the commercial version of the Starling device, which should not be used for this purpose. As a matter of fact, 11 (26%) patients were wrongly classified by Starling-24.4 at the EEXPO test. Regarding our secondary goal, we confirmed that the EEXPO test was correctly assessed if the averaging and refresh times were reduced to 8 and one seconds, respectively. In the overall population, all but 5 patients were correctly defined as “preload responders” and “preload non-responders” by the Starling-8.1 device. However, among 2 of the 3 false negatives, the ΔCI_Starling-8.1_ was close to the 5% cut-off value (respectively, 4.7% and 4.8%). This was also the case in one of the 2 false positives (5.7%). Our results suggest that bioreactance can be used to perform the EEXPO test only if the averaging and refresh times of the device are shortened, at least transiently.

Our Bland–Altman and concordance analyses showed that the estimation of the absolute value of CI by bioreactance was far from perfect. The percentage error was high, confirming previous studies [11, 35]. The Bland–Altman analysis did not provide different results for CI_Starling-8.1_ and CI_Starling-24.4_. On the contrary, the trending ability of the device was much better. In particular, the polar plot analysis of changes provided acceptable results. Interestingly, when changes were assessed during EEXPO, the trending ability of CI_Starling-8.1_ was better than that of CI_Starling-24.4_, confirming that these short-term changes were better tracked by the former version than by the latter.

Of note, the present study also contributes to the validation of the EEXPO test. The EEXPO-induced ΔCI measured by pulse contour analysis well detected preload responsiveness, which was estimated through the PLR-induced ΔCI. The AUROC was above 0.900, a level achieved only by very reliable tests and indices of preload responsiveness [24, 36]. The fact that these results were obtained in patients ventilated with a *Vt* ≤ 6 mL/kg confirms that low Vt ventilation does not make the EEXPO test unreliable, despite studies affirming the contrary [37, 38]. In addition, it confirms that in the presence of low Vt, the reliability of both PPV and SVV is limited: as shown in Table 2, no significant differences were observed between preload responders and non-responders. Of note, a limitation of the EEXPO test is that the patients must be able to sustain a rather long ventilator occlusion. In the present study, the Richmond Agitation Sedation Scale score was quite high.

### Limitations

First, we defined preload responsiveness by a positive PLR test and a fluid bolus was not infused in all the patients. However, the demonstration of PLR test reliability is likely strong enough today to allow one to consider it as a reliable surrogate of a fluid bolus [24]. Second, we investigated only a 15-s EEXPO test; a duration of 30 s has also been described [34]. With a longer EEXPO, the performances of CI_Starling-8.1_ and CI_Starling-24.4_ in tracking ΔCI might have differed less. Third, we included only hemodynamically stable patients who did not require changes in vasopressor dosage: we cannot, therefore, address the issue of whether the reliability of bioreactance could be influenced by short-term changes in afterload. In addition, sepsis was the cause of circulatory failure in most of the patients (86%). Thus, in theory, our results should apply only to this specific population. Finally, we investigated only ICU patients, though the best reliability of bioreactance has been demonstrated in normal subjects [39, 40] or in the peri-operative setting [8, 9].

## Conclusion

The Starling bioreactance device reliably detects preload responsiveness through the EEXPO test, provided that its averaging time is reduced to 8 s and its refresh time to one second.

## Supplementary Information

**Additional file 1**. Additional tables and figures.

## Data Availability

Individual, de-identified participant data are available from the corresponding author on reasonable request.

## Abbreviations

AUROC: Area under the receiver operating characteristic curve
CI: Cardiac index
CI_Starling-24.4_: Cardiac index measured by the commercial version of the Starling device (averaging time 24 seconds, refresh time 4 seconds)
CI_Starling-8.1_: Cardiac index derived through raw data analysis of the Starling device (averaging time 8 seconds, refresh time 1 second)
CI_pulse_: Cardiac index measured by the pulse contour analysis method
EEXPO: End-expiratory occlusion.
ICC: Intraclass correlation coefficient
ICU: Intensive care unit.
PLR: Passive leg raising
Vt: Tidal volume
ΔCI: Changes in cardiac index
