# Supplementary material for: Bioreactance reliably detects preload responsiveness by the end-expiratory occlusion test when averaging and refresh times are shortened
Source: Ann Intensive Care. 2021 Aug 28;11:133. doi: 10.1186/s13613-021-00920-7 (PMC8401368; doi:10.1186/s13613-021-00920-7)
Supplement: Supplementary file 1 — Additional file 1. Additional tables and figures. [file 13613_2021_920_MOESM1_ESM.docx]

Bioreactance reliably detects preload responsiveness by the end-expiratory occlusion test when averaging and refresh times are shortened

Francesco GAVELLI, MD^1,2^; Alexandra BEURTON, MD, PhD^1^; Jean-Louis TEBOUL, MD, PhD^1^; Nello DE VITA, MD^1^; Danila AZZOLINA, PhD^3^; Rui SHI, MD^1^; Arthur PAVOT, MD^1^; Xavier MONNET, MD, PhD^1^.

1 Service de médecine intensive-réanimation, Université Paris-Saclay, AP-HP, Hôpital de Bicêtre, DMU CORREVE, Inserm UMR S_999, FHU SEPSIS, CARMAS, Le Kremlin-Bicêtre, France

2 Emergency Medicine Unit, Department of Translational Medicine, Università degli Studi del Piemonte Orientale, 28100, Novara, Italy

3 Research Support Unit, Department of Translational Medicine, Università degli Studi del Piemonte Orientale, 28100, Novara, Italy

Additional file

Table of contents

[Additional file table 1 3](#_Toc74150618)

[Additional file table 2 4](#_Toc74150619)

[Additional file table 3 6](#_Toc74150620)

[Additional file figure 1 7](#_Toc74150621)

[Additional file figure 2 9](#_Toc74150622)

[Additional file figure 3 10](#_Toc74150623)

#

# Additional file table 1

Ability of the end-expiratory occlusion test to detect preload responsiveness using three different methods for measuring cardiac index in patients with Body mass index ≥ 24 kg/m^2^ and < 24 kg/m^2^

*BMI ≥ 24 kg/m^2^*

| **Variable** | **AUROC ± SE** | **Sensitivity**  **(95% CI)** | **Specificity**  **(95% CI)** | **LR +**  **(95% CI)** | **LR -**  **(95% CI)** | **Cut-off** | **p** |
| --- | --- | --- | --- | --- | --- | --- | --- |
| **EEXPO - Relative ΔCI_pulse_** | 1.000 ± 0.000 | 1.00  (0.69-1.00) | 1.00  (0.72-1.00) | - | - | 3.0% | <0.0001 |
| **EEXPO - Relative ΔCI_Starling-24.4_** | 0.591 ± 0.143 | 0.50  (0.19-0.89) | 1.00  (0.72-1.00) | - | 0.50  (0.30-0.90) | 0.1% | NS |
| **EEXPO - Relative ΔCI_Starling-8.1_** | 0.918 ± 0.063 | 0.80  (0.44-0.98) | 1.00  (0.72-1.00) | - | 0.20  (0.06-0.70) | 9.5% | <0.0001 |

*BMI < 24 kg/m^2^*

| **Variable** | **AUROC ± SE** | **Sensitivity**  **(95% CI)** | **Specificity**  **(95% CI)** | **LR +**  **(95% CI)** | **LR -**  **(95% CI)** | **Cut-off** | **p** |
| --- | --- | --- | --- | --- | --- | --- | --- |
| **EEXPO - Relative ΔCI_pulse_** | 0.957 ± 0.032 | 0.88  (0.62-0.98) | 1.00  (0.48-1.00) | - | 0.13  (0.0-0.5) | 4.1% | < 0.0001 |
| **EEXPO - Relative ΔCI_Starling-24.4_** | 0.681 ± 0.136 | 0.69  (0.41-0.89) | 0.80  (0.28-1.00) | 3.44  (0.60-20.5) | 0.39  (0.20-0.90) | 0.1% | NS |
| **EEXPO - Relative ΔCI_Starling-8.1_** | 0.905 ± 0.078 | 0.86  (0.57-0.98) | 1.00  (0.29-1.00) | - | 0.14  (0.00-0.05) | 1.5% | < 0.0001 |

AUROC: area under the receiver operating characteristic curve; EEXPO: end-expiratory occlusion; LR+: positive likelihood ratio; LR-: negative likelihood ratio; SE: standard error; 95% CI: 95% confidence interval; ΔCI_pulse_: changes in cardiac index measured through the pulse contour analysis method; ΔCI_Starling-24.4_: changes in cardiac index detected by the commercial version of the Starling device (averaging time 24 seconds, refresh time 4 seconds); ΔCI_Starling-8.1_: changes in cardiac index derived through raw data analysis of the Starling device (averaging time 8 seconds, refresh time 1 second).

Additional file

Ability of the end-expiratory occlusion test to detect preload responsiveness using three different methods for measuring cardiac index in patients without and with norepinephrine infusion.

*Without norepinephrine infusion*

| **Variable** | **AUROC ± SE** | **Sensitivity**  **(95% CI)** | **Specificity**  **(95% CI)** | **LR +**  **(95% CI)** | **LR -**  **(95% CI)** | **Cut-off** | **p** |
| --- | --- | --- | --- | --- | --- | --- | --- |
| **EEXPO - Relative ΔCI_pulse_** | 1.000 ± 0.000 | 1.00  (0.69-1.00) | 1.00  (0.48-1.00) | - | - | 3.0% | < 0.0001 |
| **EEXPO - Relative ΔCI_Starling-24.4_** | 0.700 ± 0.138 | 0.50  (0.19-0.81) | 1.00  (0.48-1.00) | - | 0.50  (0.30-0.90) | 0.1% | NS |
| **EEXPO - Relative ΔCI_Starling-8.1_** | 0.840 ± 0.110 | 0.80  (0.44-0.98) | 1.00  (0.48-1.00) | - | 0.20  (0.06-0.70) | 4.8% | 0.0019 |

*With norepinephrine infusion*

| **Variable** | **AUROC ± SE** | **Sensitivity**  **(95% CI)** | **Specificity**  **(95% CI)** | **LR +**  **(95% CI)** | **LR -**  **(95% CI)** | **Cut-off** | **p** |
| --- | --- | --- | --- | --- | --- | --- | --- |
| **EEXPO - Relative ΔCI_pulse_** | 0.977 ± 0.026 | 1.00  (0.79-1.00) | 0.91  (0.59-1.00) | 11.00  (1.70-71.30) | - | 3.3% | < 0.0001 |
| **EEXPO - Relative ΔCI_Starling-24.4_** | 0.699 ± 0.108 | 0.69  (0.41-0.89) | 0.91  (0.59-1.00) | 7.56  (1.10-50.40) | 0.34  (0.20-0.70) | 0.1% | NS |
| **EEXPO - Relative ΔCI_Starling-8.1_** | 0.921 ± 0.055 | 0.79  (0.49-0.95) | 1.00  (0.66-1.00) | - | 0.21  (0.10-0.60) | 9.5% | < 0.0001 |

AUROC: area under the receiver operating characteristic curve; EEXPO: end-expiratory occlusion; LR+: positive likelihood ratio; LR-: negative likelihood ratio; SE: standard error; 95% CI: 95% confidence interval; ΔCI_pulse_: changes in cardiac index measured through the pulse contour analysis method; ΔCI_Starling-24.4_: changes in cardiac index detected by the commercial version of the Starling device (averaging time 24 seconds, refresh time 4 seconds); ΔCI_Starling-8.1_: changes in cardiac index derived through raw data analysis of the Starling device (averaging time 8 seconds, refresh time 1 second).

# Additional file table 3

Ability of the PLR-induced changes in cardiac index obtained by the two bioreactance methods to predict preload responsiveness

| **Variable** | **AUROC ± SE** | **Sensitivity**  **(95% CI)** | **Specificity**  **(95% CI)** | **LR +**  **(95% CI)** | **LR -**  **(95% CI)** | **Cut-off** | **p** |
| --- | --- | --- | --- | --- | --- | --- | --- |
| **PLR - Relative ΔCI_Starling-24.4_** | 0.929 ± 0.039 | 0.85  (0.65-0.96) | 1.00  (0.79-1.00) | - | 0.15  (0.1-0.4) | 10% | < 0.0001 |
| **PLR - Relative ΔCI_Starling-8.1_** | 0.970 ± 0.024 | 0.92  (0.73-0.99) | 0.93  (0.66-1.00) | 12.8  (1.9-8.15.2) | 0.01  (0.0-0.3) | 15% | < 0.0001 |

AUROC: area under the receiver operating characteristic curve; LR+: positive likelihood ratio; LR-: negative likelihood ratio; PLR: passive leg raising; SE: standard error; 95% CI: 95% confidence interval; ΔCI_Starling-24.4_: changes in cardiac index detected by the commercial version of the Starling device (averaging time 24 seconds, refresh time 4 seconds); ΔCI_Starling-8.1_: changes in cardiac index derived through raw data analysis of the Starling device (averaging time 8 seconds, refresh time 1 second).

# Additional file figure 1

STARD flow-chart.

EEXPO: end-expiratory occlusion; PLR: passive leg raising; ΔCI_pulse_: changes in cardiac index detected by the pulse contour analysis method; ΔCI_Starling-24.4_: changes in cardiac index detected by the commercial version of the Starling device (averaging time 24 seconds, refresh time 4 seconds).

# Supplementary figure 2

Bland-Altman plots for measurements performed at all time points. Concordance analysis for absolute values at EEXPO_start_, EEXPO_end_, PLR_start_ and PLR_end_ for CI_Starling-24.4_ *vs.* CI_pulse_ (left) and CI_Starling-8.1_ *vs.* CI_pulse_ (right). Green dashed lines represent Bland-Altman 95% limit of agreement for repeated measurements. Light blue continuous line represents regression line.

CI_pulse_: cardiac index measured by the pulse contour analysis method; CI_Starling-24.4_: cardiac index measured by the commercial version of the Starling device (averaging time 24 seconds, refresh time 4 seconds); CI_Starling-8.1_: cardiac index derived through raw data analysis of the Starling device (averaging time 8 seconds, refresh time 1 second); EEXPO: end-expiratory occlusion; PLR: passive leg raising.

# Additional file figure 3

Polar plot analysis during PLR.

Polar Plot Analysis for relative changes in CI_Starling-24.4_ (red dots) and CI_Starling-8.1_ (blue dots) compared to the changes in CI_pulse_ during PLR.

The radial limits of the agreement have been reported. The central exclusion zone (continuous light blue circle) removes data points where the changes in cardiac index are small (less than 1.5%). The polar concordance at 30 degrees is 81% for CI_Starling-8.1_ and 86% for CI_Starling-24.4_.

CI_pulse_: cardiac index measured by the pulse contour analysis method; CI_Starling-24.4_: cardiac index measured by the commercial version of the Starling device (averaging time 24 seconds, refresh time 4 seconds); CI_Starling-8.1_: cardiac index derived through raw data analysis of the Starling device (averaging time 8 seconds, refresh time 1 second); PLR: passive leg raising.
